# Factors influencing on health-related quality of life in South Korean with chronic liver disease

**DOI:** 10.1186/s12955-018-0964-1

**Published:** 2018-07-18

**Authors:** Hyun Jin Kim, Hyeonsik Chu, Seonhye Lee

**Affiliations:** 1 Department of Quality Improvement, Hanyang University Hospital, 222-1, Wangsimni-ro Seongdong-gu, Seoul, 04763 Republic of Korea; 2 Hanyang Cell Therapy Center, Hanyang University Hospital, 222-1, Wangsimni-ro Seongdong-gu, Seoul, 04763 Republic of Korea; 3 Department of Nursing, Gyeongnam National University of Science and Technology, 33, Dongjin-ro, Jinju-si, Gyeongsangnam-do 52725 Republic of Korea

**Keywords:** Chronic liver disease, Quality of life, EQ-5D

## Abstract

**Background:**

The objective of this study was to determine health-related quality of life (HRQoL) among chronic liver disease (CLD) subjects in South Korea using EuroQol five-dimension questionnaire (EQ-5D).

**Method:**

The sample consisted of 139 subjects with CLD from the sixth Korean National Health and Nutrition Examination Survey (KNHNES VI). Data were analyzed using SPSS program for descriptive statistics, t-test, ANOVA, Scheffe’s test and hierarchical multiple regression.

**Results:**

Results indicated that marital status (*P* < 0.01), occupation (*P* < 0.01), basic livelihood security recipient status (*P* < 0.05), hepatocellular carcinoma (*P* < 0.05), subjective health status (*P* < 0.01), and depression (*P* < 0.001) were significant predictors of HRQoL. Health behaviors (alcohol intake, sleep duration) variables were insignificant.

**Conclusion:**

In conclusion, marital status, occupation, basic livelihood security recipient status (BLSRS), hepatocellular carcinoma (HCC), subjective health status (SHS), and depression were confirmed to be factors affecting the HRQoL. We should be provide to continuous monitoring and education of adequate alcohol intake for patients with CLD. Findings of this study might be used to develop community based health programs and policies for CLD.

## Background

CLD is one of the most prevalent diseases in the world. In Korea, CLD is a major disease with high prevalence along with hypertension and diabetes. According to Korea Statistics Office [1], CLD accounted for 13.3% of deaths in Korea, ranking the 8th in mortality rate in 2016. Most CLDs are caused by hepatitis B virus or hepatitis C virus, excessive alcohol consumption, fatty liver, and inflammatory reaction and healing process that are repeated for a long period of time, leading to progression to cirrhosis or liver cancer. Korea has the highest liver cancer mortality rate among OECD countries, with the highest prevalence of CLD among men in their 40s who are socially most productive. For this reason, CLD is becoming a social issue not only for the health of the individual, but also for the disease burden worldwide [2, 3].

People with CLD may suffer from specific complications of cirrhosis such as ascites, hepatic encephalopathy, and variceal bleeding. Moreover, fatigue, joint pain, skin itching, loss of appetite, depression, loss of libido, and problem of memory are associated CLD. Furthermore, CLD is linked to job loss, impaired functioning, anxiety, and low self-esteem that severely affect the quality of life [4–6]. In a previous study, it has been emphasized that a combination of psycho-social aspects and treatment is an important factor that determines the quality of life of CLD patients [4]. Therefore, the HRQoL of patients with CLD is an important outcome that should be considered for the treatment and prognosis of such patients [7].

The HRQoL of patients with chronic illness is affected by SHS and disease progression. CLD is not only a physical problem. It also affects a comprehensive range of physical and psycho-social aspects such as self-care, daily life activities, anxiety, and depression of patients [8, 9]. Previous studies have reported that age, gender, economic status, depression, self-efficacy, types of CLD, stage of disease, family history of CLD, and comorbidities are influencing factors of HRQoL [2, 4, 8, 10, 11]. However, the HRQoL of patients with CLD is not as well studied as other chronic diseases. In particular, nursing does not pay particular attention to factors that affect the quality of life of patients with CLD [12]. In South Korea, a few studies have compared HRQoL of CLD patients to that of patients with other chronic diseases [13–15].

It is necessary to grasp the quality of life of CLD by using the Korea National Health and Nutrition Examination Survey (KNHANES) data for the management of appropriate chronic diseases. KNHANES is conducted every year to evaluate the current state of the public’s health and nutritional status, the health vulnerable group, the effectiveness of health policy and project, and the quality of life by disease [16]. Therefore, the aim of this study was to identify factors influencing the HRQoL of patients with CLD in Korea using data from the 6th KNHANES. Results of this study could be used as a basis to develop nursing interventions and policies that contribute to improved quality of life for CLD subjects.

## Methods

### Data and study design

We performed a cross-sectional study using data from the sixth KNHANES. To identify factors influencing the HRQoL of subjects with CLD, we obtained raw data of the sixth KNHANES which was conducted for household members older than 1 year. The KNHANES was composed of health, nutrition, and examination questionnaires. The Korea Centers for Disease Prevention and Control (KCDC) conducts a KNHANES annually by sending trained surveyors to investigate a representative sample using a computer-assisted personal interviewing technique. We have obtained approval to use the raw data from the KCDC homepage.

There were 18,034 adults over 19 years old out of 22,948 people in the sixth KNHANES. For this study, 139 from 140 CLD subjects currently suffering from hepatitis B, hepatitis C, liver cirrhosis, and HCC were included after excluding one unfaithful respondent. In order to confirm the number of samples necessary for executing the regression analysis, we used G*power 3.1.5.program to calculate the size of subjects using intermediate effect size of .15, significance level of .05, power analysis of .80, and 10 predictive variables. A total of 118 subjects were judged as an appropriate number of samples.

### Ethical considerations

KNHANES was an annual survey conducted by the Research Ethics Review Committee of the KCDC (No. 2013-07CON-03-4C, No. 2013-12EXP-03-5C, No. 2015–01-02-6C). All personal identification information was deleted from the data before analysis. Participants in this survey provided their informed consent.

### Variable measurement

#### Health related quality of life (HRQoL)

HRQoL was measured using the EQ-5D (EuroQoL-5 dimension) developed by European EuroQoL Group [17] to measure overall health. EQ-5D consisted of mobility, self-care, usual activities, pain/discomfort, and anxiety/depression. Responses in each dimension were divided into three levels coded as ‘no problems-1,’ ‘moderate problem-2,’ and ‘severe problem-3’. In this study, we used the model developed by the KCDC [18] which applied weighted values for HRQoL in the Korean context to calculate the EQ-5D index score ranging from − 1 point (severe problem) to + 1 point (no problem).

#### Socio-demographic characteristics

For socio-demographic characteristics, age, gender, marital status, educational level, occupation, and BLSRS were surveyed.

#### Health status

Health status was measured by SHS, depression, perceived stress, degree of obesity, comorbidity, types of CLD, and current treatment for CLD. *SHS* was assessed using the question, ‘How do you think about your health status in usual?’. Participants were asked to choose one in a 5-points scale: very unhealthy (1 point), unhealthy (2 point), medium (3 point), healthy (4 point), and very healthy (5 point), with the higher score indicating the healthier. For data analysis of this study, answers of very healthy and healthy were categorized as ‘good health’ while all other answers were categorized as ‘poor health’. In practical terms, SHS is readily measured using a single- item question, and is often included in health surveys and as an outcome in many studies [19]. *Depression* was defined as having experienced depression in their daily life for more than 2 weeks in a year. It was measured as: ‘not depressed’ or ‘experienced of depression’. *Perceived stress* was classified as ‘yes’ if participant felt it in their everyday life ‘very much’ or ‘feel a lot’. All other responses were reclassified as ‘no.’ *Degree of obesity* was measured by body mass index (BMI) which was calculated by dividing body weight (kg) by the square of height (m^2^). BMI < 18.5 was classified as ‘underweight,’ ≤18.5 ∼ < 23.0 as ‘normal weight,’ ≤23.0 ∼ < 25.0 as ‘overweight,’ and ≥ 25.0 as ‘obese’ [20]. The metric was categorized as either ‘underweight/normal’ or ‘overweight/obese’. *Comorbidity* was operationalized as the number of chronic diseases diagnosed by a physician (i.e., hypertension, hyperlipidemia, diabetes, strokes, myocardial infarction, angina etc.). It was categorized as ‘without comorbidity’ or ‘with comorbidities’. The *types of CLD* were consisted of hepatitis B, hepatitis C, liver cirrhosis and HCC. *Current treatment* for CLD was categorized as either ‘yes’ or ‘no’.

#### Health behaviors

Health behaviors such as alcohol intake, smoking, regular health check-up, cancer screening, regular exercise, and sleep duration per day were measured. Those variables were categorized as either ‘yes’ or ‘no’. *Alcohol intake* was a single question about drinking in the past year. ‘How often do you alcohol intake in the past year?’ The answer is Likert 5 point scale, ‘no alcohol intake,’ ‘one time in a month,’ ‘about once in a month,’ ‘about 2-4 times a month,’ ‘around 2-3 times a week,’ and ‘more than 4 times a week.’ In this study, ‘No’ was used for ‘no alcohol intake’ and ‘Yes’ for ‘the others response.’ *Smoking* was a question ‘Do you smoke now?’ In the choices of responses were ‘daily smoking,’ ‘sometime smoking,’ ‘no smoking,’ and ‘smoked in the past but not currently smoking.’ In this study, ‘no smoking,’ and ‘smoked in the past but not currently smoking’ were reclassified as ‘non-smokers,’ and ‘the others’ were ‘smokers.’ The questions of *regular health check-up* and *cancer screening* were ‘Did you have regular health check-up during the recent two years?, and ‘Did you have cancer screening during the recent two years?’ Subjects answered the questions ‘Yes’ or ‘No.’ *Regular exercise* was criteria by Korea  Ministry of Health and Welfare [21] as a regular activity criterion for adults and elderly. The criteria was evaluated to ‘have regular exercise’ when ‘middle level activity’ for 2 h 30 min or more or ‘high level activity’ for 1 h 15 min or more, and the other exercise were reclassified to ‘no regular exercise.’ Sleep duration per day was categorized as ‘appropriate sleeping duration’ or ‘inappropriate sleeping duration.’ The criteria were developed by Hirshkowitz et al. [22]. Appropriate sleeping duration was changed by age. It was that young adults (18-25 years) were 6–11, adults (26–64) were 6–10, and older adults (≥ 65) were 5–9 h.

### Data analysis

Data were analyzed using SPSS (Version 22, IBM, Armonk, NY, USA). Socio-demographic characteristics of subjects, health status, health behavior, and EQ-5D index were analyzed using frequency and percentage. Differences in EQ-5D index by socio-demographic characteristics, health status, and health behavior were identified using t-test, analysis of variance and posthoc-test was applied Scheffe’s test. Hierarchical multiple regression analyses were performed to predict the EQ-5D index. Dummy variables were marital status, occupation, BLSRS, SHS, depression, comorbidity, alcohol intake, and sleep duration per day. The Durbin-Watson statistic was 2.100, indicating no autocorrelation in residuals. Tolerance ranged from 0.413 to 0.916. Variance inflation factor ranged from 1.091 ∼ 2.421, indicating an absence of multicollinearity, thus satisfying basic assumptions of regression. After eliminating cases where normalized residuals were larger than the absolute value (10 cases), we performed a rank regression analysis for 129 subjects. Hierarchical multiple regression analysis was performed using Model I (socio-demographic characteristics), Model II (health status), and Model III (health behaviors).

## Results

### HRQoL according to variables

Table 1 shows differences of mean in HRQoL according to socio-demographic characteristics, health status, and health behaviors of Korean CLD patients. The most frequent socio-demographic characteristics were ‘45 ∼ 64 years’ (56.9%), ‘male’ (62.6%), ‘with partner’ (81.2%), ‘over college degree’ (28.8%), ‘with occupation’ (59.0%), and ‘basic livelihood security non-recipient’ (88.5%). The mean age of subjects was 55.3 ± 12.7 years. The HRQoL was found to differ significantly according to age (*P* < 0.05), marital status (*P* < 0 .01), education level (*P* < 0.01), occupation (*P* < 0.01), and BLSRS (*P* < 0.05). The most frequent characteristics of health status were ‘unhealthy’ (85.6%), ‘no depression’ (82.6%), ‘no perceived stress’ (75.4%), ‘normal/under-weight’ (64.0%), ‘no comorbidity’ (55.4%), and ‘current treatment for CLD’ (62.6%). The types of CLD were ‘hepatitis B’ (57.6%), ‘hepatitis C’ (5.8%), ‘liver cirrhosis’ (24.5%), and ‘HCC’ (12.2%). The HRQoL showed significant differences according to SHS (*P* < 0.001), depression (*P* < 0.05), type of CLD (*P* < 0.001), and comorbidity (*P* < 0.05). The most frequent health behaviors were ‘alcohol drinker’ (51.4%), ‘non-smoker’ (76.8%), ‘doing regular health check-up’ (76.8%), ‘doing cancer screening’ (70.5%), ‘regular exercise’ (54.0%), and ‘appropriate sleep duration’ (79.9%). The HRQoL also showed significant differences according to alcohol intake (*P* < 0.05) and sleeping duration (*P* < 0.01). The most frequent types of comorbidity were hypertension 31.7% and the mean BMI of subjects was 24.04 ± 3.05 (not in the table).

**Table 1:** HRQoL according to Characteristics of CLD subjects in South Korea (*N* = 139)

Characteristics		Categories	n(%)	EQ-5D Index	*t*-value (Scheffe’s test)
				M ± SD	
Socio-demographic	Age(years) (M ± SD: 55.3 ± 12.7)	< 45^a^	27(19.4)	0.98 ± 0.04	3.414^*^ (a > c)
		45 ∼ 64^b^	79(56.9)	0.90 ± 0.19	
		≥ 65^c^	33(23.7)	0.89 ± 0.12	
	Gender	Male	87(62.6)	0.92 ± 0.17	0.780
		Female	52(37.4)	0.90 ± 0.14	
	Marital status^e^	Living with partner	108(81.2)	0.93 ± 0.16	2.860^**^
		Living without partner	25(18.8)	0.83 ± 0.15	
	Education level	≤Elementary school^a^	37(26.6)	0.83 ± 0.24	6.024^**^ (c,d>a)
		Middle school^b^	24(17.3)	0.92 ± 0.11	
		High school^c^	38(27.3)	0.94 ± 0.11	
		≥College^d^	40(28.8)	0.96 ± 0.07	
	Occupation	Yes	82(59.0)	0.95 ± 0.11	3.307^**^
		No	57(41.0)	0.86 ± 0.20	
	Basic livelihood security recipient	Yes	16(11.5)	0.80 ± 0.19	−2.621^*^
		No	123(88.5)	0.93 ± 0.15	
Health status	Subjective health status	Good	20(14.4)	0.99 ± 0.17	5.453^***^
		Poor	119(85.6)	0.90 ± 0.17	
	Depression^e^	Yes	24(17.4)	0.82 ± 0.25	−2.083^*^
		No	114(82.6)	0.93 ± 0.12	
	Perceived stress^e^	Yes	34(24.6)	0.86 ± 0.24	−1.739
		No	104(75.4)	0.93 ± 0.12	
	Degree of obesity	Underweight/normal	89(64.0)	0.91 ± 0.18	−0.732
		Overweight/obese	50(36.0)	0.93 ± 0.11	
	Comorbidity	Yes	62(44.6)	0.88 ± 0.19	−2.242^*^
		No	77(55.4)	0.94 ± 0.12	
	Types of CLD	Hepatitis B^a^	80(57.6)	0.94 ± 0.10	6.973^***^ ( a,c,d>b)
		Hepatitis C^b^	8(5.8)	0.71 ± 0.44	
		Liver cirrhosis^c^	34(24.5)	0.87 ± 0.13	
		HCC^d^	17(12.2)	0.94 ± 0.13	
	Current treatment for CLD	Yes	87(62.6)	0.92 ± 0.15	0.444
		No	52(37.4)	0.91 ± 0.17	
Health behaviors	Alcohol intake^e^	Yes	71(51.4)	0.95 ± 0.11	2.555^*^
		No	67(48.6)	0.88 ± 0.19	
	Smoking	Yes	32(23.2)	0.89 ± 0.23	−1.025
		No	106(76.8)	0.92 ± 0.13	
	Regular medical check-up^e^	Yes	106(76.8)	0.92 ± 0.13	1.130
		No	32(23.2)	0.88 ± 0.23	
	Cancer screening test	Yes	98(70.5)	0.92 ± 0.13	0.993
		No	41(29.5)	0.89 ± 0.21	
	Regular exercise	Yes	64(46.0)	0.93 ± 0.12	1.092
		No	75(54.0)	0.90 ± 0.19	
	Sleep duration	Appropriate	111(79.9)	0.93 ± 0.11	2.733^**^
		Inappropriate	28(20.1)	0.84 ± 0.28	

*Note. M* mean, *SD* standard deviation, *HCC* Hepatocelluar carcinoma, Categories with superscripts(age, educational level and types of CLD), were group name for analyzing Scheffe's test. A>B : A was higher mean than B.

^*^P < 0.05, ^**^ *P* < 0.01, ^***^P < 0.001

^e^missing value was excluded

### Factors affecting HRQoL

To identify factors affecting HRQoL of patients with CLD, hierarchical regression analysis was performed (Table 2). In Model I, among socio-demographic characteristics (age, marital status, education level, occupation, BLSRS), marital status (*P* < 0.05), occupation (*P* < 0.01) and BLSRS (*P* < 0.05) were found to have significant associations, with an explanatory power of 25.6% (*P* < 0.001). When health status variables were entered (Model II), HCC (*P* < 0.05), SHS (*P* < 0.01) and depression (*P* < 0.001) were significant associated, with explanatory power of 39.8% (*P* < 0.001). When health behaviors (alcohol intake, sleep duration) variables were entered (Model III), results were insignificant.

**Table 2:** Factors influencing HRQoL (*N* = 129)

Predictor	Model I		Model II		Model III	
Variables	ß	SE	ß	SE	ß	SE
(Constant)	.911	.045	.847	.051	.817	.067
1. Socio-demographic factors						
Age	− 244^**^	.001	−.192	.001	−.170	.001
Marital status(ref: Living without partner)	.201^*^	.019	.160^*^	.017	.159^*^	.017
Education level(ref: ≤Elementary school)						
Middle school	.112	.022	.078	.020	.076	.020
High school	.007	.021	.013	.019	.006	.019
≥College	−.129	.018	−.142	.016	−.144	.017
Occupation(ref: No)	.231^**^	.015	.243^**^	.014	.221^**^	.014
Basic livelihood security recipient(ref: Yes)	.187^*^	.025	.146	.023	.163^*^	.023
2. Health status factors						
Types of hepatic disease(ref:Hepatitis C)						
Hepatitis B			.014	.033	.012	.033
Liver cirrohosis			−.135	.017	−.109	.017
HCC			.188^*^	.022	.187^*^	.022
Subjective health status(ref: Poor)			.218^**^	.018	.226^**^	.018
Depression(ref: Yes)			.292^***^	.017	.273^***^	.017
Medical co-morbidity(ref: Yes)			.045	.014	.040	.014
3. Health behavior factors						
Alcohol intake (ref: Yes)					.125	.014
Sleep duration(ref: inappropriate)					.030	.017
R^2^(R^2^ change)	.297		.459(.162)		.472(.013)	
Adjusted R^2^	.256		.398		.402	
F(p)	7.288(<.001)		7.506(<.001)		6.729(<.001)	

*Note. ref* referent group, ß = regression coefficients, *SE* standard error, *HCC* Hepatocelluar carcinoma

^*^ *P* < 0.05, ^**^ *P* < 0.01, ^***^ *P* < 0.001

## Discussion

The mean of EQ-5D in Korean of CLD was 0.91, similar to the quality of life in the general population [23, 24], but higher than those with chronic obstructive pulmonary disease [13, 25] and CLD patients in Italy [26]. These differences might be due to variations in data collection. To better understand the quality of life of patients with CLD, an understanding of the characteristics and severity of disease is needed.

The HRQoL of CLD in Korea was found to be significantly different according to age, marital status, education level, occupation, BLSRS. Chinese CLD patients [27] have also shown significantly difference according to age and marital status. For US chronic hepatitis C patients [28], HRQoL is different according to gender, education level, and marital status. In Brazilian CLD patients [5], HRQoL is different according to economic status. However, in Germany [4], HRQoL of CLD patients has no association with age, gender, or economic status. These results of previous studies were inconsistent. This might be due to differences in the type of liver disease, research measurement tools, and so on. CLD is a disease prevalent in men in their 40s to 50s who are most active in economic activities. They might face problems such as disintegration of family and declining productivity of society. CLD not only affects individuals, but also the society. Therefore, it is necessary to understand factors influencing the HRQoL of Korean CLD patients through repeated researches. Spousal supports have positive effect on family livelihood and patient’s quality of life [27]. Therefore, supporting groups for CLD spouses are needed. Poor people are vulnerable groups who have economic difficulties arising from repeat hospitalization [29–31] and the lack of self-management for health [32]. Therefore, a multidisciplinary nursing intervention program that considers various characteristics of CLD with low HRQoL is needed.

In this study, 85.6% of subjects recognized that they were unhealthy. Although depression was only reported by 17.4% of subjects, it was found to be a factor influencing the HRQoL. CLD patients with comorbidity showed low HRQoL in this study. However, previous studies have revealed inconsistent results with low [4] or high [28] quality of life depending on the type of liver disease. HRQoL is an important factor that should be evaluated for patients with chronic diseases. It can provide a balance between clinical and patient outcomes [33]. CLD subjects often have negative emotions such as psychological sensitivity and concern due to fatigue, ascites, and esophageal varices [34–37]. Subject with hepatitis C were showed the lowest HRQoL. This result was also revealed in the precedent study [33, 38]. 60% of hepatitis C has converted to liver cirrhosis and HCC [39], side effects such as fatigue, depression, impotence etc. possessed by interferon which is a treatment have difficulties in home and work life [38]. Especially, the prevalence rate is as low as 0.6–0.8%, but 50–80% of the infected people are chronic condition, resulting in a great economic and psychological burden which is personal or national aspects. In Korea, it is managed as a third-party national infectious disease [40]. This burden of disease management seems to have lowered the HRQoL of the hepatitis C subjects. CLD needs various supportive care, including information and educational, practical, physical, psychological needs, and patient care support (support group, etc.) [41]. In this study, HRQoL of HCC was higher than previous studies [42, 43]. The QoL of HCC patients reported worse physical, emotional and functional QoL, but better social/family QoL compared with the general population [44]. This data was extracted from the KNHANES. This survey collected the data on subjects who can conduct a one-hour questionnaire and physical examination alone [45]. The HRQoL of HCC was closely related to liver function, tumor stage, recurrent HCC, fatigue, pain, nausea, and performance status [43]. Therefore, careful attention should be paid to the extended interpretation of this result, and repeated research is needed.

As health behavior variables, alcohol drinking and sleep duration showed significant differences in the HRQoL of CLD patients without affecting it. Alcohol drinking is a major predictor of liver disease [28]. Continuous drunk monitoring [46] and management program of adequate drinking are necessary to reduce the deterioration in HRQoL of CLD patients and prevent the increase of medical expenses. Similar to a previous study [47], CLD subjects with less than 6 h of sleep appeared to have poor quality of life. Adult and older adults were not recommended less than 6 h of sleep. Sleep, like diet and exercise, is a vital part of physical, cognitive, and emotional health [22]. Therefore, it will be necessary to have a repeated study to verify that affecting factors the quality of life such as alcohol and sleep of patients with CLD. Symptoms of fatigue and sleep disorder can reduce their quality of life. CLD occurred in those with abnormal circadian rhythm in a previous study [48]. Therefore, we need to assess factors affecting sleep disorder to provide sleep nursing intervention. Also, qualitative research is required to understand the health behavior affecting the HRQoL of CLD patients.

This study has several limitations. First, we had limitations that to assess depression, comorbidity and stress, validated methods are not used (ex. Beck depression inventory, Hamilton scale, Charlson Comorbidity Index, Perceived Stress Scale). The severity of CLD was not assessed by Child-Pugh or MELD (Model for End-Stage Liver Disease). We did not evaluated the patients had ascites or edema, because this was a secondary analysis of the KNHANES data. Second, there were restrictions in analyzing the causal relationship of various influence factors in the secondary analysis of the KNHANES data. Caution is needed when expanding and interpreting our research results due to the lack of prior research measured with EQ-5D. Despite these limitations, the result from this study came from a sample based on nationally representative data. We evaluated HRQoL of CLD through EQ-5D. The results of this study can be compared other national or disease of HRQoL and used as basis data to develop community based health programs for patients with CLD.

## Conclusion

In this study, we analyzed the sixth KNHANES in order to grasp factors influencing the HRQoL of patients with CLD in Korea. As a result, marital status, occupation, BLSRS, SHS, depression and HCC were confirmed as affecting factors, health behavior variables were not significant. Alcohol intake has a negative effect on liver function. But, the HRQoL of those who intake an alcohol is significantly higher. We should be provide to continuous monitoring and education of adequate alcohol intake for patients with CLD. Future research will require a longitudinal study and a cohort approach to obtain a deeper understanding of health behavior and causal relationship with HRQoL in Korean CLD. And HRQoL studies of CLD patients should be undertaken through application of validated measurement tool.

## Abbreviations

BLSRS: Basic livelihood security recipient status
CLD: Chronic liver disease
EQ-5D: EuroQol five-dimension
HCC: Hepatocelluar carcinoma
HRQoL: Health-related quality of life
KCDC: Korea Centers for Disease prevention and Control
KNHANES: Korea National Health and Nutrition Examination Survey
SHS: Subjective health status
